# Predictive value of net water uptake for early neurological deterioration after mechanical thrombectomy in acute ischemic stroke with large vessel occlusion

**DOI:** 10.3389/fneur.2025.1649063

**Published:** 2025-10-07

**Authors:** Min Kuang, Junying Li, Jian Wang, Guangwen Chen, Chao Luo

**Affiliations:** ^1^Department of Radiology, West China School of Medicine, Sichuan University, Sichuan University Affiliated Chengdu Second People’s Hospital, Chengdu, China; ^2^Department of Neurology, West China School of Medicine, Sichuan University, Sichuan University Affiliated Chengdu Second People’s Hospital, Chengdu, China

**Keywords:** computed tomography, net water uptake, early neurologic deterioration, mechanical thrombectomy, stroke

## Abstract

**Purpose:**

To investigate whether Net Water Uptake (NWU) can predict early neurological deterioration (END) after mechanical thrombectomy (MT) in acute ischemic stroke with large vessel occlusion (AIS-LVO).

**Materials and methods:**

We retrospectively analyzed consecutive patients with AIS-LVO who underwent MT. Patients were categorized into the END group and the non-END group based on whether END occurred. NWU was an imaging parameter to quantify the water uptake capacity of brain tissue and measured on admission non-contrast computed tomography (NCCT). Early edema progression rate (EPR) was determined as the ratio of NWU and time from symptom onset to baseline imaging. Then, the baseline characteristics were subsequently collected. Variable and multiple regression analyses were performed to explore independent risk factors for END. Finally, receiver operating characteristic (ROC) curves were constructed to evaluate the predictive value of NWU for END.

**Results:**

A total of 158 patients were included. The median NWU, admission National Institutes of Health Stroke Scale (NIHSS) and EPR in END group was 10.1% (IQR: 6.8–15.4), 16(IQR: 15–19) and 0.087% (IQR: 0.038–0.187). Respectively, the non-END group was 6.8% (IQR: 0–10.9), 13(IQR: 8–17) and 0.043% (IQR: 0–0.096). Compared with the non-END group, the END group had higher NWU (*p* = 0.004), higher admission NIHSS score (*p* = 0.001), and higher EPR (*p* = 0.006); multiple logistic regression showed that NWU (odds ratio [OR], 1.084; 95% confidence interval [CI], 1.004–1.171, *p* = 0.039) and admission NIHSS score (OR, 1.124; 95%CI, 1.032–1.224; *p* = 0.007) were independent risk factors for END. ROC curve showed that NWU had a moderate predictive ability for END. The area under the ROC curve (AUC) was 0.665 (95%CI, 0.561–0.770). The AUC of admission NIHSS score was 0.687 (95%CI, 0.698–0.776). NWU combined with admission NIHSS score had the highest predictive value for END, with an AUC of 0.739 (95%CI, 0.648–0.831).

**Conclusion:**

The NWU was an independent predictor of END and increased NWU is associated with END in patients with AIS-LVO after MT. Similarly, the admission NIHSS score was also an independent predictor. The combination of NWU and the admission NIHSS score achieves the strongest predictive ability for END.

## 1 Introduction

Stroke remains a common cause of human mortality and disability and represents a substantial burden on global public health. Both the incidence and prevalence of stroke have shown an increasing trend in recent years (1). Furthermore, the most common type is acute ischemic stroke (AIS), accounting for approximately 70–80% of strokes (2). AIS caused by large vessel occlusion (AIS-LVO) is characterized by rapid onset, severe clinical manifestations, a high mortality rate, and a relatively low treatment success rate (3). Currently, intravenous thrombolysis and mechanical thrombectomy (MT) are the main treatment options for AIS-LVO (1), of which MT has been proven to be an effective intervention. MT can bring great benefits to patients, especially for the occluded part in the internal carotid artery and the proximal segment of the middle cerebral artery (1, 4).

However, some patients still experience poor prognosis despite timely MT (5, 6) and even develop early neurological deterioration (END) (7), which means the progressive aggravation of neurological function impairment in patients during the early stage of stroke. It occurs in approximately 20–40% of patients with ischemic stroke, significantly increasing the risk of mortality and medical expenses and bringing a heavy economic burden on society and families (8, 9). Consequently, identifying both imaging and non-imaging biomarkers associated with END has become a critical challenge in recent years.

Magnetic resonance imaging (MRI) and computed tomography (CT) perfusion imaging dominate the primary field of imaging biomarkers for END, whereas non-contrast computed tomography (NCCT) biomarkers are relatively uncommon. However, NCCT is an essential imaging modality for stroke assessment (1) and has advantages such as easy availability, low cost, and short imaging time. Therefore, a new imaging biomarker based on NCCT, Net Water Uptake (NWU), has recently attracted our attention. NWU is a CT-based imaging biomarker that can quantify the degree of brain tissue edema by calculating the percentage change in density between the ischemic and normal region on NCCT image. Currently, it has been investigated in several studies on the diagnosis and treatment of AIS (10–14). Therefore, we attempted to explore whether NWU and other risk factors can predict END in patients with AIS-LVO after MT. The goal was to help identify END as soon as possible and investigate the potential value of NWU based on NCCT in the application of stroke.

## 2 Materials and methods

### 2.1 Patients

This study retrospectively analyzed consecutive patients with AIS-LVO treated in the Department of Neurology at our hospital from January 2021 to December 2024. Patients were divided into two groups, END and non-END, on the basis of whether END occurred. END was defined as an increase in the National Institute of Health Stroke Scale (NIHSS) score by ≥2 points or dyspraxia score by ≥1 point within 7 days after admission (15, 16). The NIHSS scores were jointly evaluated and determined by two senior neurologists. Inclusion criteria were as follows: (1) patients with AIS-LVO in the anterior circulation (internal carotid artery or M1 segment of the middle cerebral artery) confirmed by digital subtraction angiography (DSA); (2) at least 18 years of age; (3) MT was conducted within 24 h of symptom onset, with successful recanalization criteria identified by a modified thrombolysis in cerebral infarction grade of at least 2b; (4) NCCT, CT Angiography (CTA), and CT perfusion (CTP) were performed upon admission. Exclusion criteria were as follows: (1) other large vessel lesions; (2) bilateral vascular abnormalities; (3) hemorrhage and encephalomalacia confirmed by NCCT before MT; (4) AIS-LVO complicated with other lesions in the brain; (5) other serious diseases such as malignant tumors and systemic lupus erythematosus; (6) incomplete imaging and clinical data; (7) obvious image artifact interference and inaccurate NWU segmentation region; and (8) failed recanalization after MT.

### 2.2 Patients’ demographic and clinical information

The following factors were included: age; sex; time from symptom onset to baseline imaging; NIHSS score at admission; presence of hypertension, diabetes, atrial fibrillation, coronary heart disease, rheumatic heart disease, and high cholesterol level; stroke etiology (smoking for >6 months, >10 cigarettes/day); and alcohol consumption history (continuous alcohol consumption for >1 year with daily alcohol intake >50 g).

### 2.3 Image acquisition

The patients underwent NCCT, followed by CTP and CTA at admission using a 256-section CT scanner (Revolution; GE Healthcare, Chicago, IL, United States). NCCT: 120 kV, 180–400 mA, 5 mm, from the foramen magnum to the vertex. CTA: 100 kV, 250–400 mA, 5 mm; 50 mL of iodinated contrast medium (Lopamidol, Jiangsu Hengrui Pharmaceuticals, Lianyungang, Jiangsu, China) was injected with a 40 mL saline flush performed from the aortic arch to the vertex. CTP: 80 kV, 100–150 mA, 5 mm; 30–40 mL of iodinated contrast medium (Lopamidol, Jiangsu Hengrui Pharmaceuticals) was injected at a rate of 5 mL/s, with 30 consecutive spiral scans of the whole brain.

### 2.4 Image analysis and data collection

**Figure 1:**
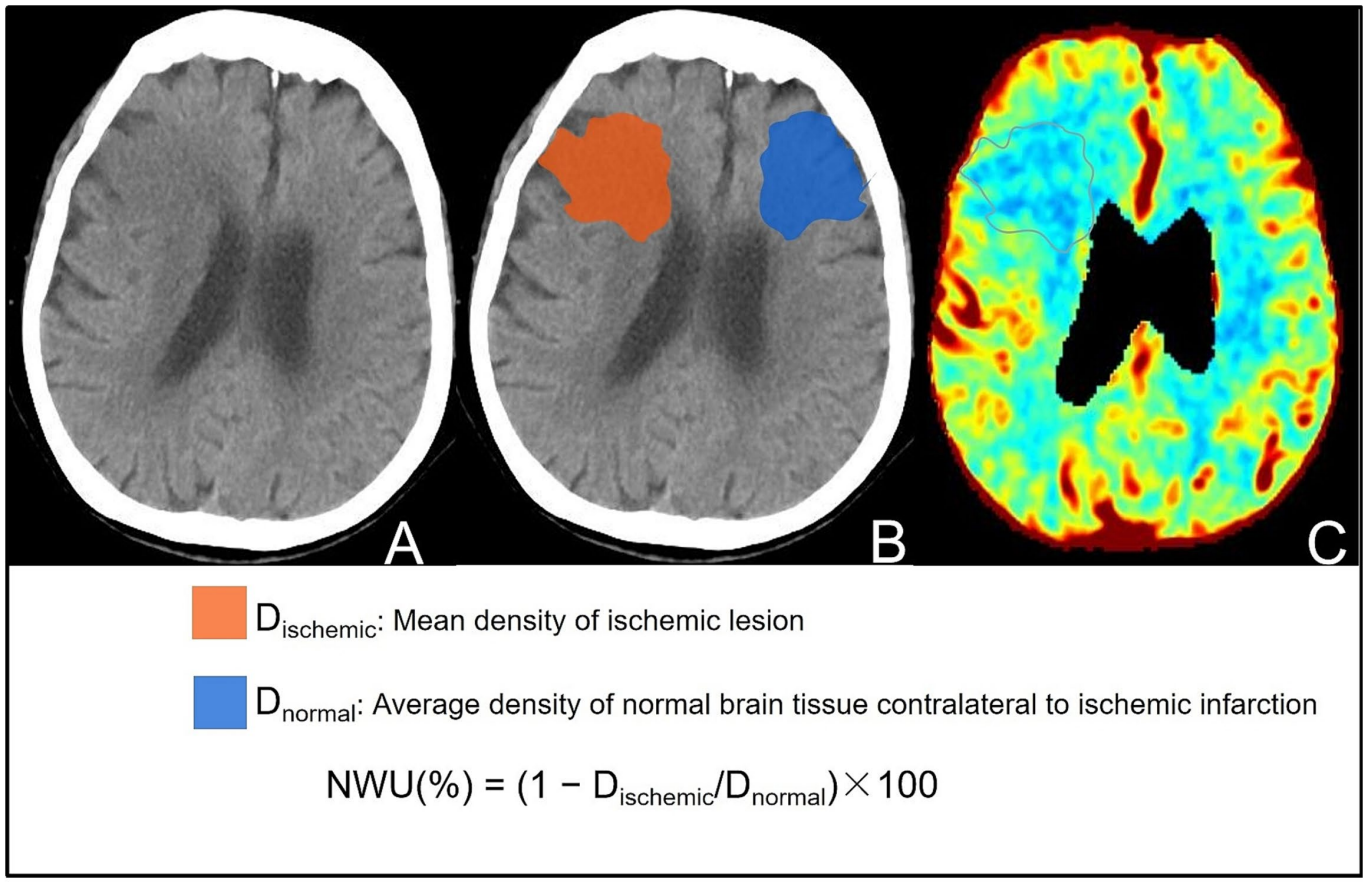
Visual representation of the steps for calculating NWU on the baseline NCCT images of the same patient. **(A)** NCCT image showing patchy slightly low attenuation near the lateral ventricle of the right frontal lobe. **(B)** Segmented lesion area (red: ischemic lesion area; blue: opposite normal brain tissue). The NWU was calculated based on the average CT value of the segmented area, and the calculation formula is shown in the figure. **(C)** CBV map verifying the accuracy of the ischemic partition, showing consistency between reduced CBV and the partitioned area.

Automatic analysis software (uAI, United Imaging, Shanghai, China) was used to segment the ischemic lesion (region of interest [ROI]) and automatically calculate the NWU using NCCT on admission, which was blindly verified by two senior neuroradiologists (with >10 years of experience). The ROI was matched and compared with the ischemic area shown in the cerebral blood volume (CBV) map (perfusion value, 0–2 mL/100 mL) of the CTP, and cases with inaccurate segmentation regions were eliminated. The NWU calculation method is illustrated in Figure 1. Early edema progression rate (EPR) was calculated by dividing NWU by the time from symptom onset to baseline imaging (17).

### 2.5 Statistical analysis

Statistical analysis was performed using the SPSS 26 software package (IBM, Armonk, NY, United States). *p-*value <0.05 was considered statistically significant. Mean (standard deviation) or median (interquartile range) were used for continuous variables. Student’s *t*-tests or Mann–Whitney *U* test were used on the basis of data distribution. Categorical variables were described as frequencies (%) and analyzed using Fisher’s exact test or *χ*^2^ test. Multivariate logistic regression was used to identify independent risk factors. Finally, receiver operating characteristic (ROC) curve analysis was used to evaluate the predictive effect of independent risk factors for END.

## 3 Results

### 3.1 Patient characteristics

**Figure 2:**
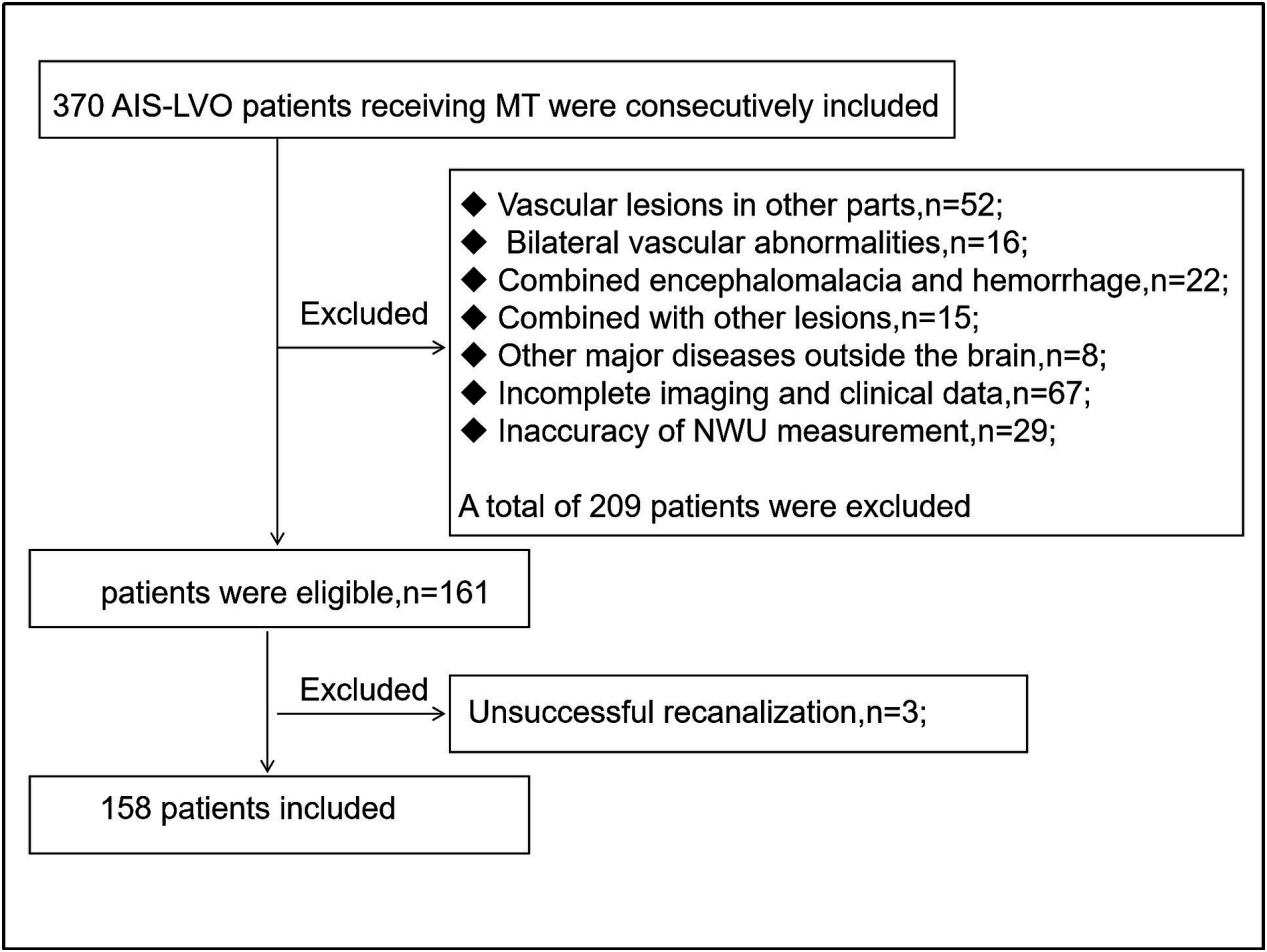
Flow chart of inclusion and exclusion criteria.

**Figure 3:**
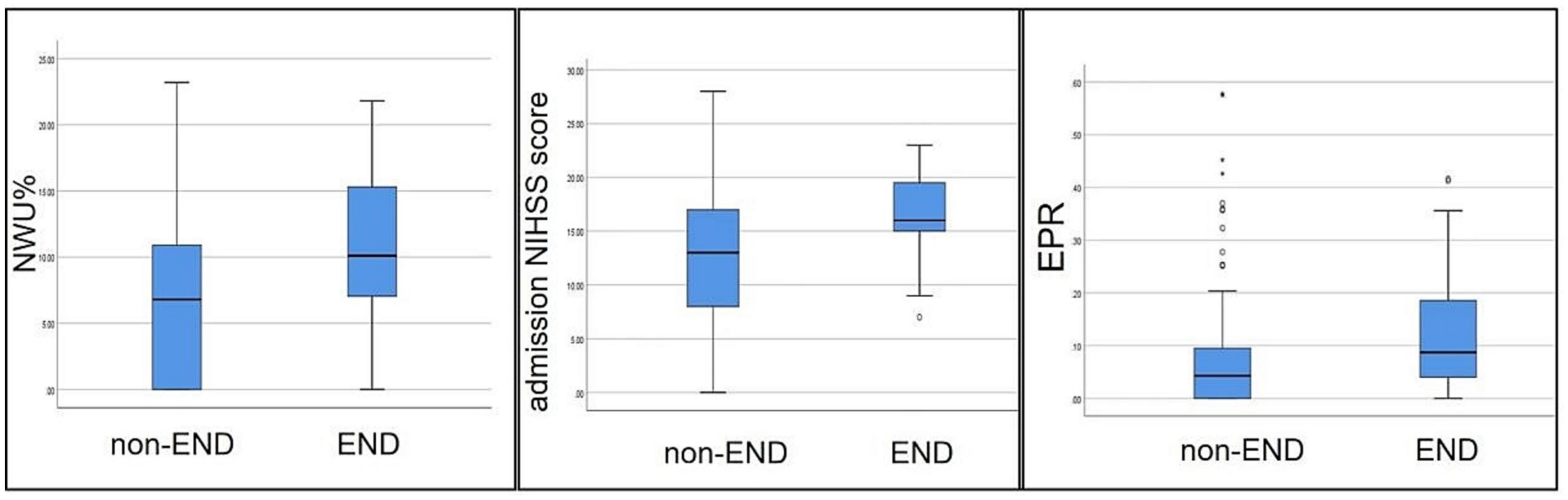
Comparison of NWU, admission NIHSS scores, and EPR between the two groups. Each box plot visually shows that the END group scores are higher than those of the non-END group (*p <* 0.05).

A total of 158 patients were included in the final analysis (Figure 2), of which 126 (79.7%) were in the non-END group and 32 (20.3%) in the END group. The main baseline patient characteristics and pairwise comparisons of various factors are shown in Table 1. No significant differences were observed in age, sex, alcohol consumption, smoking, hypertension, diabetes, atrial fibrillation, coronary heart disease, rheumatic heart disease, or high cholesterol level between the two groups (*p* > 0.05). Significant differences were observed in NWU, admission NIHSS score, and EPR between the two groups (*p* < 0.05); the values in the END group were significantly higher than those in the non-END group (Figure 3). Eighteen cases of malignant cerebral edema (MCE) or symptomatic intracerebral hemorrhage (sICH) occurred after MT, and one case was observed in the non-END group.

**Table 1:** Characteristics in patients with the END group and the non-END group.

Characteristics	END group (*n* = 32)	Non-END group (*n* = 126)	*P*-value
Age (years), median (IQR)	77(65–84)	72(65–82)	0.202
Male, *n* (%)	14(43.8%)	58(46.0%)	0.845
Smoking, *n* (%)	6(18.8%)	27(21.4%)	0.813
Drinking, *n* (%)	3(9.4%)	18(14.3%)	0.661
Hypertension, *n* (%)	17(53.1%)	63(50.0%)	0.844
Diabetes, *n* (%)	11(34.4%)	24(19.0%)	0.062
Atrial fibrillation, *n* (%)	8(25.0%)	38(30.2%)	0.620
Coronary heart disease, *n* (%)	4(12.5%)	11(8.7%)	0.755
Rheumatic heart disease, *n* (%)	2(6.3%)	11(8.7%)	0.924
High cholesterol, *n* (%)	0(0.0%)	5(3.9%)	0.317
Admission NIHSS score, median (IQR)	16(15–19)	13(8–17)	0.001
NWU, median (IQR)	10.1(6.8–15.4)	6.8(0.0–10.9)	0.004
EPR, median (IQR)	0.087(0.038–0.187)	0.043(0.0–0.096)	0.006

END, early neurologic deterioration; IQR, inter quartile distance; NIHSS, national institute of health stroke scale; NWU, net water uptake; EPR, early edema progression rate.

### 3.2 Multivariate analysis identifies independent predictors of END

Multiple logistic regression analysis showed that after adjusting for confounding factors, NWU (odds ratio [OR], 1.084; 95% confidence interval [CI], 1.004–1.171; *p* = 0.039; Table 2) and admission NIHSS score (OR, 1.124; 95%CI, 1.032–1.224; *p* = 0.007; Table 2) remained independent predictors of END.

**Table 2:** Multivariable logistic regression analysis for prediction of END.

Variables	OR	95% CI	*P*-value
Admission NIHSS score	1.124	1.032–1.224	0.007
NWU	1.084	1.004–1.171	0.039
EPR	2.185	0.058–82.740	0.673

END, early neurologic deterioration; NIHSS, national institute of health stroke scale; NWU, net water uptake; EPR, early edema progression rate; CI, confidence interval; OR, Odds Ratio.

### 3.3 NWU and admission NIHSS score in predicting END

**Figure 4:**
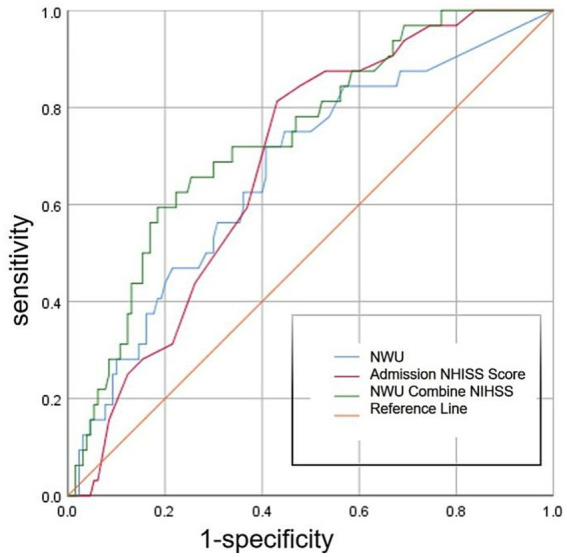
ROC curve showing the diagnostic performance of three different methods for predicting END.

ROC curve analysis showed (Figure 4) that the optimal cutoff value of NWU for predicting the occurrence of END was 8.0%, with a sensitivity of 71.9%, specificity of 58.7%, and area under the ROC curve (AUC) of 0.665 (95%CI, 0.561–0.770; *p* = 0.004; Table 3). The optimal cutoff value of the admission NIHSS score was 15 points, with a sensitivity of 81.3% and specificity of 57.1% (Figure 4). The AUC of admission NIHSS score was 0.687 (95%CI, 0.698–0.776; *p* = 0.001; Table 3). NWU combined with admission NIHSS score had the highest predictive ability for END, with an AUC of 0.739 (95%CI, 0.648–0.831; *p* = 0.000; Table 3 and Figure 4), sensitivity of 59.4%, and specificity of 81.7%.

**Table 3:** The comparative analysis for predicting END.

Variables	AUC	95% CI	*P*-value
Admission NIHSS score	0.687	0.698–0.776	0.001
NWU	0.665	0.561–0.770	0.004
NWU combine admission NIHSS score	0.739	0.648–0.831	0.000

END, early neurologic deterioration; NIHSS, national institute of health stroke scale; NWU, net water uptake; AUC, area under ROC curve; CI, confidence interval.

## 4 Discussion

In our study, we identified multiple factors that influenced END in AIS-LVO of the anterior circulation. The results demonstrated that NWU was an independent predictor of END; patients with a higher NWU were more likely to experience END even after successful recanalization using MT. Additionally, the admission NIHSS score was an independent factor, and the combination of the two biomarkers exhibited the strongest predictive ability for END. These findings may assist clinicians in identifying patients at high risk of END prior to MT. Furthermore, they are not only valuable for the prevention and management of END, but also beneficial for identifying patients who are most likely to benefit from MT.

The NWU is an independent factor influencing END because it is closely related to the etiology of END. It is well established that the pathogenesis of END involves multiple factors, encompassing a range of mechanisms such as cerebral edema, symptomatic intracerebral hemorrhage, recurrent stroke, and systemic complications (14), with sICH and MCE being two dominant factors (14, 18). Moreover, many studies have demonstrated that NWU can quantify the degree of cerebral edema and predict MCE (13, 19–21), as well as predict sICH (22, 23). These intrinsic connections enabled NWU to serve as a predictor of END. Furthermore, our analysis revealed that among the 32 patients with END, 18 had MCE or sICH after MT (18/32), whereas only 1 case (1/126) was observed in the non-END group, which is consistent with the above explanation. Additionally, one study indicated that NWU is an independent predictor of 90-day poor functional outcomes (24), which complements our findings that END is strongly associated with poor 90-day outcomes (7, 8, 25). Therefore, NWU shows promise as a predictive marker for END and clinical outcomes in patients with AIS. In clinical practice, it may play an important role in the comprehensive management, diagnosis and treatment of stroke in the future. For example, Minnerup et al. (10) demonstrated that NWU can accurately estimate the stroke onset within 4.5 h, enabling timely thrombolysis in patients with unknown onset time. Ghozy et al. (26) reported that NWU enables prediction of MCE early and helps identify patients who may benefit from decompressive craniectomy.

Moreover, compared with other biomarkers related to END, NWU has some unique advantages. At present, related research has garnered much attention, and numerous studies have previously explored the prediction of END in stroke involving a wide array of factors, including imaging and non-imaging biomarkers. Non-imaging biomarkers, such as blood pressure, blood glucose levels, plasma atherosclerosis index, glycated albumin, erythrocyte sedimentation rate, and platelet-to-lymphocyte ratio (27–32), they are vary and are easily influenced by other factors in the human body compared with imaging biomarkers. For imaging biomarkers of END, most research has focused on MRI and CTP imaging, such as unfavorable cerebral venous outflow, cerebral blood volume, amide proton transfer weighting, arterial spin labeling, and susceptibility-weighted imaging (33–35). Although these imaging biomarkers hold significant potential for improving the prediction and management of END, their clinical application is not widespread owing to high costs, lengthy examination times, need for specialized equipment, and contraindications associated with MRI. In contrast, NWU can address the aforementioned shortcomings, and with the development of artificial intelligence, NWU measurements will be more rapid, accurate, and standardized. It is worth noting that NWU not only offers significant advantages in predicting END, but also demonstrates broader applicability in various aspects of stroke. Example, DWI/FLAIR mismatch is commonly used to estimate lesion age in patients with an unknown onset time (36), however, its reliability is limited by the subjective interpretation of the evaluator. In contrast, NWU provides a quantitative assessment with superior inter-rater consistency, making it a more objective and practical tool in emergency decision-making (37). Similarly, arterial spin labeling (ASL) has been established as an effective biomarker for assessing tissue reperfusion. Nevertheless, its clinical application remains constrained, particularly in patients with AIS presenting with high NIHSS scores, and NWU may offers a feasible and unaffected alternative in such cases (38).

Additionally, our results indicated that the NIHSS score at admission was another independent predictor of END, which is consistent with previous studies. A systematic review (8) demonstrated that the NIHSS score at admission quantifies the severity of neurological deficits. Furthermore, early neurological abnormalities detected using the NIHSS score are critical indicators of potential complications. Several clinical studies have also suggested that the NIHSS score at admission is an independent risk factor for END in patients with cerebral infarction (39–41). Moreover, NIHSS score at admission is associated with ineffective recanalization or poor outcomes (24). It is evident that both NWU and admission NIHSS scores were related to END. Our results also showed that the combined ability of the two in predicting END was higher than that of each alone. Importantly, these two biomarkers are readily available and easily accessible. Patients with stroke can be assessed using the two biomarkers within half an hour of admission, even in community hospitals. This approach is expected to help in the rapid clinical identification of patients at high risk of END. However, further investigation is warranted.

This study has the following limitations. First, this study may exhibit a selection bias because we employed a retrospective research design, and the measurement of historical data may not be completely standardized. Second, the measurement of NWU through artificial intelligence automated measurement software, which may cause the segmentation of interest region to be asymmetric and accurate. In order to ensure the accuracy of NWU measurements as much as possible, we used strict exclusion criteria to reduce measurement errors, and double-blind manual proofreading of NWU measurements. Third, the sample size of the END group is relatively small; relevant cases need to be collected in the future to expand the sample size and improve the accuracy of the research results. Finally, our study participants were limited to patients with AIS-LVOs in the anterior circulation; therefore, the generalizability of our findings may be limited.

## 5 Conclusion

In conclusion, this study demonstrated that higher NWU and admission NIHSS scores were associated with the occurrence of END after MT in patients with AIS-LVO. The combination of these two biomarkers may provide important information for the early identification of END and may also assist in the clinical screening of patients who benefit more from thrombectomy. Therefore, further prospective studies with larger sample sizes are warranted.

## Data Availability

The raw data supporting the conclusions of this article will be made available by the authors, without undue reservation.
